# 

**Published:** 1888-01

**Authors:** 


					﻿J-ATxTTJJYRY, 18&8.
OLD SERIES
VoL xxv
/OS
NEW SERIES
Vol. IV.-No. II.

HEDIGAL^lJRt®
JOURNAL
■ 2).

W.F.WESTMOREL AND. N®
H.VM.M1LLER. M.D.LL .DJR
JAMES A.GRAY. M.D.
PUBLISHED BY JAMES P.HARRISON&CGH
Atlanta,ga- JI
SUBSCRIPTION: 52.50 A YEAR.
				

